# Musculoskeletal Disorders’ Classification Proposal for Application in Occupational Medicine

**DOI:** 10.3390/ijerph18158223

**Published:** 2021-08-03

**Authors:** Pablo Monteiro Pereira, João Amaro, Bruno Tillmann Ribeiro, Ana Gomes, Paulo De Oliveira, Joana Duarte, João Ferraz, João Santos Baptista, José Torres Costa

**Affiliations:** 1 Associated Laboratory for Energy, Transports and Aeronautics, (LAETA/ROA), Faculty of Medicine, University of Porto, 4200-319 Porto, Portugal; prof.monpe@outlook.com (P.M.P.); zecatoco@sapo.pt (J.T.C.); 2 Institute of Public Health, University of Porto, 4200-319 Porto, Portugal; amaro.jpc@gmail.com (J.A.); paulo.oliveira.ortopedia@gmail.com (P.D.O.); 3 Occupational Safe and Health Department, IMTEP/PETROBRAS, Macaé 27913-350, Brazil; brunotillmann@gmail.com; 4 Occupational Safe and Health Department, Ria Blades/Siemens Gamesa, 3840-346 Vagos, Portugal; hannalleh@gmail.com; 5 Associated Laboratory for Energy, Transports and Aeronautics, (LAETA/PROA), Faculty of Engineering, University of Porto, 4200-465 Porto, Portugal; jasduarte@fe.up.pt (J.D.); ferraz.jhm@gmail.com (J.F.)

**Keywords:** classification, WRMSD, MSD, occupational diseases, musculoskeletal disorders, occupational health

## Abstract

Occupational-specific classifications of musculoskeletal disorders (MSD) are scarce and do not answer specific clinical questions. Thus, a specific classification was developed and proposed, covering criteria applicable to daily clinical activity. It was considered that the disorder development process is the same across all work-related MSDs (WRMSDs). Concepts of clinical pathology were applied to the characteristics of WRMSDs pathophysiology, cellular and tissue alterations. Then, the correlation of the inflammatory mechanisms with the injury onset mode was graded into four levels (MSDs 0–3). Criteria of legal, occupational and internal medicine, semiology, physiology and orthopaedics, image medicine and diagnostics were applied. Next, the classification was analysed by experts, two occupational physicians, two physiatrists and occupational physicians and one orthopaedist. This approach will allow WRMSD prevention and improve therapeutic management, preventing injuries from becoming chronic and facilitating communication between occupational health physicians and the other specialities. The four levels tool relate aetiopathogenic, clinical, occupational and radiological concepts into a single classification. This allows for improving the ability to determine a WRMSD and understanding what preventive and therapeutic measures should be taken, avoiding chronicity. The developed tool is straightforward, easy to understand and suitable for WRMSDs, facilitating communication between occupational physicians and physicians from other specialities.

## 1. Introduction

The classification of musculoskeletal disorders (MSDs), recognised as disorders of tendinous, muscular and articular origin [1], has always been important in the medical field, having been studied for over 100 years [2]. The classification of MSDs embraces a wide variety of disorders, including tendonitis, tendinosis, degenerative joint lesions, arthrosis, and neural involvement by tendon compression. Between 1986 and 2020 alone, and to better understand and more effectively classify MSDs, 74 independent classifications were created in sports. From those 74 classifications, 72 are presented in a systematic review by Hamilton et al. (2015) [2], and two other were published between 2017 and 2020 [3,4].

According to Bahr et al. (2020) [3], the most widely used clinical classification is based on the “onset” of the injury′s occurrence, whether sudden or repetitive. However, its practical application in identifying some injuries is simplistic and leads to confusion [3], particularly concerning traumatic injuries on injured tissues or gradual injuries. In the last decade, five muscle injury classifications have been updated in sports to improve the MSD classification [4,5,6,7,8].

However, even when updated, MSD classifications in sports cannot be adequately applied in other situations, such as MSDs with an occupational source (work-related musculoskeletal disorders—WRMSDs [9]). This problem occurs in the clinical context when non-acute MSDs are observed, given that most sport classifications are linked to acute or traumatic injuries [10].

According to the pathophysiological context, all MSDs develop from two types of inflammatory mechanisms: acute and chronic. In Table 1, its description, clinical and biochemical findings, and results of the inflammatory process in cell/tissue damage are demonstrated, according to these two inflammatory mechanisms [11,12,13].

During the MSD pathophysiological analysis in professional environments, it is observed that injuries mainly result from excessive loads or repetitive tasks, with characteristics of over-stretching, compression, friction, ischemia and overexertion [12].

**Figure 1:**
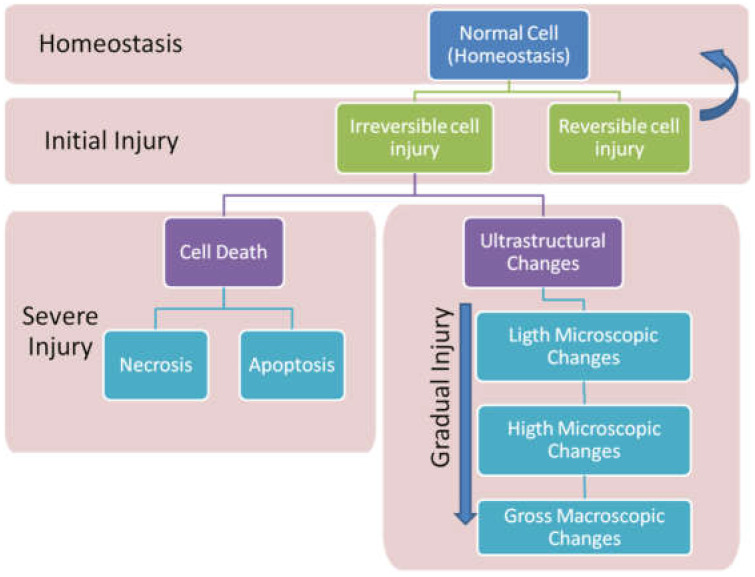
Cellular and tissue regeneration process after injury.

According to Kumar et al. [11], the continual execution of physical activities while the tissue is still inflamed hinders the interruption of the inflammatory cycle. This overload will promote inflammatory progression, leading the inflammation to a state of chronicity caused by structural and morphological changes (Figure 1), making cell damage irreversible.

Thus, considering the pathophysiological concept as a reference, it is possible to verify that the existing classifications within the scope of occupational medicine are limited [14]. Consequently, they can promote an incomplete assessment of the injury, compromising its proper prevention and treatment.

In an occupational medicine context, there are two major classifications for WRMSDs: the classification of the International Labour Organization (ILO) [15] and the type developed by Bernard et al. [16], published by the National Institute for Occupational Safety and Health (NIOSH).

According to ILO, WRMSDs can be classified as occupational diseases, with a codification or as occupational accidents [15]. Any other injury not related to these two categories is considered a work-aggravated disease, according to the International Classification of Diseases (ICD) [17]. 

According to ICD, 10th edition [17], the classifications referring to MSDs receive a code for classification, which is not applicable for use in a clinical therapeutic context [17].

According to NIOSH classification [16], the mechanisms of occurrence of WRMSDs injuries consider the risk factors recognised as occupational. Nevertheless, the significance of Bernard and colleagues’ classification [16], in the work context, is restricted to identifying the risk factors that can cause occupational diseases recognised by ILO. However, it does not present information based on injury physiopathology. Despite this lack of information, the use of Bernard’s classification was widespread in the occupational health and safety context and the work ergonomics area [18]. Furthermore, it allowed integration of the risk factors. However, its use in occupational medicine is limited because, regardless of the risk factors, workers’ health needs to associate a correct diagnosis so that therapy and preventive intervention can be well guided.

The European Agency for Safety and Health at Work (EU-OSHA) defines “WRMSDs when the disorder is caused or aggravated mainly by work and the effects of the immediate environment in which work is performed” [19]. Nevertheless, while this classification is vital in legal matters, it has little applicability in daily practice.

Thus, the significance of existing classifications is recognised. However, it is also concluded that they cannot adequately cover the peculiarities of MSDs in an occupational environment. This limitation makes it challenging to adopt effective therapeutic and preventive measures. In this context, it is necessary to overcome the lack of a specific classification for WMSD that meets occupational medicine’s diagnostic and therapeutic needs. So, this work aimed to develop a proposal for the classification of MSDs in the work context, through aetiopathogenic concepts capable of achieving increased practical effectiveness, allowing the prevention of WRMSD to have better therapeutic management and facilitate communication between occupational health physicians and those of other specialities.

## 2. General Methodology of Tool Development

Clinical pathology concepts were applied to the characteristics of MSD pathophysiology and cellular and tissue changes (Table 1). The criteria established for the new classification involved the inflammatory mechanisms and their characterisation. According to the onset, action mechanisms, risk factors involved, clinical signs and symptoms were complementary findings at ultrasound (USG) imaging and magnetic resonance (MR) imaging. Then, the inflammatory mechanisms were correlated with the mode of lesion onset-sudden or gradual and, thus, graded into four classification levels: MSD-0–MSD-3. After defining this grading scale, all other criteria were adapted to the four pre-established levels. Subsequently, legal medicine and occupational medicine criteria were applied to the mechanisms of injury and risk factors. The requirements for the description of signs and symptoms, injury location, time of recovery, and return to activity were evaluated using the medical clinic, semiology, physiatry and orthopaedics concepts. Furthermore, the identification of acute and chronic injuries was carried out according to image and diagnostic medicine criteria.

After applying the concepts to all specialities, all tables were reviewed by specialists: two physiatrists and occupational physicians, two occupational physicians and one orthopaedist.

Thus, after addressing each particular aspect, the results were summarised into one table. In total, five partial tables were created. Ultimately, all five tables were, in turn, synthesised into a single classification table.

Finally, the results were tested using those MSDs recognised as occupational by the ILO and compared with the ICD-10 classification.

## 3. Tool Development

It is understood that the processes are continuous, with the possibility of complete or incomplete tissue regeneration without healing (Figure 1). Therefore, the first analysis of the classifications comes from existing concepts and criteria according to clinical pathology (Table 1), distinguishing between acute and chronic inflammatory processes [11,12,13].

Then, the inflammatory mechanisms defined in Table 1 were correlated with the development of MSD, allowing for the distribution of the classification into four MSD levels (0–3), as shown in Table 2.

According to the forensic medicine criteria, Table 3 presents the previously defined scale levels (MSD-0 to MSD-3) relating them to tissue injury mechanisms and the risk factors identified in the workplace. In this assessment, the risk factors were divided into individual (as bone misalignments or muscle imbalances), physical (like vibration or impacts), ergonomic (mainly factors related to the biomechanics of movement) and operational (risks associated with working tasks) [12,16,18,20].

The correlations between MSD scales 0–3 and clinical symptoms, like as pain (visual analogical pain scale [21,22]) and injury zone and treatment (discussed in the internal medicine, orthopaedics, physiatry and medical semiology literature) also used in other international classifications, are shown in Table 4 [3,5,6,8,11,12,23,24,25,26,27,28].

The relationship between the MSD 0–3 scale and radiological findings is shown in Table 5. The results are presented descriptively and narratively in this report, supported by previous classifications recognised in the speciality literature [4,5,6,8,11,28,29,30].

## 4. Results

After using the different classification methods and the criteria recognised by different specialities, the tables were combined using the MSD 0–3 scales and all other studied criteria, producing a single classification table representing a summary of the process (Table 6).

The developed classification was tested for applicability purposes, comparing the classification of MSD 0–3 with occupational musculoskeletal disease codes recognised by ILO and ICD-10 codes (Table 7).

**Figure 2:**
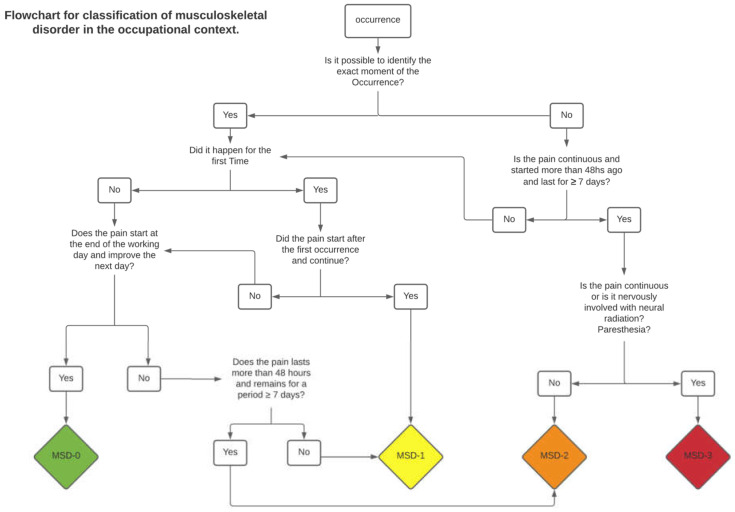
Flowchart for clinical decision in Occupational Context.

In order to simplify the clinical application of the scale, a decision flowchart was developed (Figure 2). This flowchart can be an important visual tool for daily clinical use. It can be directly applied as long as there is a perfect understanding of the defined criteria for each classification level. Detailed classification criteria are presented in Table 6.

## 5. Discussion

Considering WRMSD prevalence in approximately 60% of all work-related complaints [16], developing a classification for specific application to these diseases is an important issue. This requirement is primarily for clinical practice because it enables appropriate care management for patients and staff, improving well-being at work [15,18,19,31,32].

Considering the classifications of MSDs presented between 1896 and 2020 in the scope of sports, Hamilton et al. (2015) [2] chronologically divided these classifications into (a) “Era Clinic” (1900–1980); (b) “Era of the Image” (1985–2000), and (c) “Modern Era” (from the 2000s). However, despite the vast literature on MSD classifications presented during this period in the scope of sports, they are not fully applicable in the work context [2,3,4,5,6,7,8,33].

Therefore, typically in the work context, both ILO and ICD-10 classifications are used. Nevertheless, they are of little relevance in occupational clinical practice and do not provide criteria for better disorder management. Thus, the importance of these classifications is limited to the statistical purposes of data collection because they allow the existence of a single language between countries, as observed in the EU-OSHA annual reports [34].

According to ILO, WRMSDs are recognised as (a) “occupational accident: an unexpected and unplanned occurrence, including acts of violence, arising out of or in connection with work which results in one or more workers incurring a personal injury, disease or death” (Resolution concerning statistics of occupational injuries (resulting from occupational accidents)-1988- ISCO 88, adopted by the 16th International Conference of Labour Statisticians (October 1998)); (b) WRMSD is “[...] any disease contracted as a result of an exposure to risk factors arising from work activity” [15] and “[...] disease contracted as a result of an exposure over a specific period of time to risk factors arising from work activity” [19]. 

MSDs not present in the ILO′s list of occupational diseases [15] are referred to as MSD aggravated by work, that is, resulting from work tasks. However, it is not possible to fit them correctly into the work context. They are often considered natural diseases aggravated by work activities that do not describe the MSD occurrence mechanism.

The ILO and ICD-10 classifications do not describe the MSD occurrence mechanism. They also do not state whether the disorder occurred suddenly or gradually, nor do they define whether there is a specific type of therapeutic intervention or whether it is necessary to remove the worker from their work. Thus, these classifications do not allow for the best therapeutic guidance because they do not classify multiple criteria.

The recognition and determination of a work-related disease appear to be of financial interest to states, the employer and the employee who has suffered the injury, and they are also relevant to social security services [35,36]. The recognition that a worker′s injury or illness is professional can allow the use of this information to adjust activities and change the tasks. However, this recognition also shows that the prevention of MSDs was not enough [37,38] and that there was incorrect management of the initial lesion, allowing its evolution to chronicity.

The most important for occupational medicine is preventing and treating workers′ MSD thoroughly [39,40], allowing reversible MSD to be adequately managed, avoiding worsening and chronicity.

Therefore, descriptively divided into four levels (MSD 0–3), the developed tool is easy to understand, enabling the inclusion of any WRMSD, present or not in the ILO list. Thus, it is essential to integrate aetiopathogenic, clinical, occupational and radiological concepts into a single classification, improving the ability to determine WRMSD and understanding what preventive and therapeutic measures should be adopted.

The proposed classification facilitates communication between clinical and occupational physicians. This approach also demonstrates to other professionals in the occupational health and safety area the importance of inflammatory processes being diagnosed and treated early in the disease, preventing its progression to chronicity.

## 6. Conclusions

Reporting to the occupational context, the existing ILO and ICD-10 classifications for MSDs are not particularly useful in health prevention and promotion in the scope of occupational medicine. They are limited to classifying the disease, not predicting any intervention. Thus, its application does not point to intervention paths that may occur when the disorder has already established itself and is irreversible. These classifications are applicable in the context of data collection but are not very functional in medical practices.

Therefore, a classification that includes criteria applicable clinically and allows for better management of the events is essential to prevent a reversible MSD from becoming irreversible.

In the clinical practice of evaluating WRMSD, considering the inflammatory criteria for the proper removal of the employee helps to prevent diseases and to promote health at work. Therefore, such an evaluation has repercussions on workers′ health, the company′s operating environment and the employee′s socioeconomic context. Thus, the return or permanence of a worker in a given occupational task, with an inflammatory process that still exists (active), is a critical measure that must be avoided.

The developed tool can improve WRMSD management, enhance communication between medical professionals of different specialities and increase understanding of the disorder process for other work and professional industrial areas.

Despite being aware that each WRMSD has specificity related to risk factors, the disorder development process remains the same. This is precisely the purpose of the classification presented in this study, which used the previous MSDs concepts and classifications in developing the proposed assessment tool. The usefulness of the developed tool goes beyond classification. It improves understanding of the disorder, identifying the time of the intervention and the need to prevent injuries or their worsening.

Future studies are required to analyse specific WRMSD recovery times, disease prevention, work-task protocols and the hours necessary for job turnover considering the specificities of WRMSDs with work tasks.

## Figures and Tables

**Table 1:** Adapted classification criteria based on injury pathophysiology and tissue changes [11,12,13].

Classification	Description	Clinical Signs	Cytokine	Duration Time	Outcomes Cellular/Tissue
Acute Inflammation	− Small vessels dilation. − Increased microvascular permeability. − Leukocytes migration: the neutrophils that predominate in the first 6 to 24 h are gradually replaced by macrophages from 24 to 48 h after injury.	− Pain − Fever − Edema − Blush	TNF IL-1 IL-6 IL-17 Prostaglandins Bradykinin Reactive oxygen species (ROS)	24–48 h	− Complete recovery − Reversible cell function − Death cell − Progressive chronic inflammation
Transition Period	Between 48 h and 7 days, features of acute and chronic inflammation can be found. This period can be called the transition period, in which the diagnosis of the injury as acute or chronic is not clear.				
Chronic Inflammation	− Long-lasting response starting 48 h after injury and lasting for weeks or months. In this phase, inflammation, tissue damage, and recovery attempts coexist in different combinations. − In most chronic inflammatory reactions, the dominant cells are macrophages and T lymphocytes.	− Pain − Atrophy	IL-12 INF- IL-17	≥7 days	− Fibrosis − Loss of function − Perhaps even tissue breakdown may occur. − Granulation tissue − Neuropathic pain − Neural fibrosis − Anxiety − Depression

**Table 2:** Criteria according to lesion pathophysiology and onset mode.

Classification		Criterion		
MSD Type	Onset Mode	Inflammation	Tissue	Tissue Regeneration and Outcome
0	Late	Acute	Healthy	Complete tissue regeneration (regeneration without irreversible cellular changes)
1	Sudden	Acute	Healthy	Complete tissue regeneration without fibrosis
2	Sudden	Chronic	Healthy or Altered	Fibrosis/cell death/progressive chronic inflammation
3	Gradual	Chronic	Altered	Fibrosis/loss of function/tissue granulation Neuropathic pain/neural fibrosis

**Table 3:** Classification according to criteria of injury mechanism and risk factors inherent to activities.

Classification		Criterion		
		Mechanism/Risk Factors		
MSD Type	Onset Mode	Mechanical Action	Mechanism of Tissue Injury/Tissue Integrity	Risk Factor
0	Late	− Tissue trauma due to a tolerable effort	Alteration of homeostasis with a mild inflammatory response	Absent
1	Sudden	− External direct trauma − Tissue trauma due to excessive effort	Traumatic, generating an episode of acute inflammation in healthy tissue	Physical Operational
2	Sudden	− External direct trauma − Tissue trauma due to excessive effort	A new episode of acute inflammation in tissue previously damaged or inflamed but not fully regenerated.	Individual physical Ergonomic Operational
3	Gradual	− Trauma in the inflamed or fibrous tissue that may occur with low-energy effort	Repeated episodes of inflammation over unregenerated tissue, maintaining a continuous process of chronic inflammation.	Individual physical Ergonomic Operational

**Table 4:** Classification according to criteria for defining signs and symptom regeneration time.

Classification		Criterion			
		Clinical	Anatomical	Treatment	
MSD Type	Onset Mode	Signs and Symptoms	Injury Zone	Rest	Rehabilitation
0	Late	Mild pain, which worsens with movement, self-resolving for up to 48 h.	Ligaments/tendons Muscles	No need	No need
1	Sudden	Severe pain at the time of injury, progressive, disabling or not, remaining for days. Muscular contracture, pain on intense palpation, sometimes diffuse, local oedema, hyperaemia, presence of hematoma, pain on movement, decreased range of motion.	Ligaments/tendons Joints Muscles, Bones	Need	Need
2	Sudden	Acute, constant inflammatory pain worsens with movement, temporarily incapacitating and may remain for days to weeks. During palpation or free movement, moderate to severe pain may or may not have oedema and haematoma, decreased range of motion due to fear.	Ligaments/tendons Joints Muscles Bones	Need	Need
3	Gradual	Chronic inflammatory pain, of moderate to low intensity, with constant loss of function. Present during tasks, requiring the use of daily medication to control pain. It can worsen with task movement and decrease at rest, often related to paraesthesia.	Ligaments/tendons Joints Muscles Bones Nerves	Need	Need

**Table 5:** Classification according to Radiological findings criteria (Adapted) [2,3,5,6].

Classification		Criterion
MSD Type	Onset Mode	Radiology/Complementary Examinations (USG/RM)
0	Late	Slight exudate oedema.
1	Sudden ^b^	“Usually, when necessary, it can be positive for fibre breakage in high-resolution MRI. Intramuscular haematoma. Oedema (exudate). When partial fibre breakage in high-resolution MRI. Intramuscular haematoma, fibres, disorganised and thin, surrounded by haematoma and peripheral fluid. When complete rupture: MRI: Complete discontinuity of muscle fibres, haematoma and retraction of muscular extremities. USG: Comparable to MRI” [5]
2	Sudden ^b^	Positive when there is a dislocation or partial or complete rupture of the fibres, probably including some retraction. It may have oedema and haematoma. Bone remodelling, tendon calcification processes fibres, disorganised and thin, surrounded by haematoma and peri-fascial fluid.When complete rupture on MRI: “Complete discontinuity of muscle fibres, haematoma and retraction of muscular extremities. USG: Comparable to MRI” [5]
3	Gradual	Positive for the degenerative disorder, often including some ligamentous retraction, atrophy and dysplasia. One may have oedema and haematoma, bone remodelling, tedious calcification processes.

^b^ According Chan et al. (2012) [5].

**Table 6:** Classification process summary.

Classification	Description	Keywords
MSD 0	− Are MSDs related to Late-onset muscle pain, considered the pain and stiffness felt in the muscles several hours to days after unusual or strenuous exercise? The pain is felt more intensely 24 to 72 h after the activity or task. − Acute inflammatory process − They are self-resolving in 72 h, without the need for further examinations. − ICD-10: They are considered non-specific myalgia coded as M79.1. − ILO: They do not have criteria to be considered for occupational diseases. − USG/MRI: No changes or minor intramuscular oedema − Risk factors: Absent	Late Acute inflammation Healthy tissue Self-resolving
MSD 1	− Traumatic MSD, with injury to the muscle-tendon unit due to excessive force or SUDDEN extreme stretching, with the possibility of rupture of muscle fibres or tendon. Local haematoma, severe pain at the time of the injury, progressive, disabling or not, pain on movement, decreased motion range. − They need therapeutic intervention with ice, NSAID in place, and, in case of rupture, surgical treatment. − Acute inflammatory process − Necessary removal from work while there is an inflammatory process, necessary rehabilitation in surgical cases and muscle strengthening to return functionality. − ICD-10: There is a code depending on the injury. − ILO: Work accident − USG/MRI: Normally, positive findings acute inflammation, Intramuscular haematoma, surrounded by haematoma. When rupture: Discontinuous muscle fibres (partial or complete), rupture site is hyper-vascularised and altered in echogenicity, haematoma, without retraction of muscular extremities. − Risk Factors: physical, operational, individual	Sudden Acute inflammation Healthy tissue Clearance required Necessary therapy Rehabilitation only in cases of disruption
MSD 2	− Traumatic MSD, with injury to the muscle-tendon unit due to excessive force or sudden extreme stretching, in tissue with scar alteration or an existing inflammatory process. − They need therapeutic intervention with ice, NSAID in place, and, in case of rupture, surgical treatment. − Acute inflammatory process − Necessary removal from work while there is an inflammatory process, necessary rehabilitation in surgical cases and muscle strengthening to return functionality. − ICD-10: There is a code depending on the injury. − ILO: Work accident − USG/MRI: Normally, positive findings acute inflammation, Intramuscular haematoma, surrounded by haematoma. When rupture: Discontinuity of muscle fibres (partial or complete), haematoma and retraction of muscular extremities. − Risk factors: physical, operational, ergonomic	Sudden Acute inflammation Altered tissue Clearance required Pain therapy Functional rehabilitation
MSD 3	− Gradual progression MSD, with injury to the muscle-tendon unit due to excessive force or excessive stretching, in tissue with scar alteration or an existing inflammatory process, chronic, permanent inflammatory process, injury with associated nervous impairment. − Need therapeutic intervention with ice, NSAIDs, therapies for pain control. − Chronic inflammatory process. − Necessary removal from work, change of function, necessary rehabilitation, muscle strengthening to return strength, return to functional capacity. − ICD-10: There is a code depending on the injury. − ILO: Occupational illness or illness aggravated by work. − USG/MRI: Positive for degenerative diseases, often including some ligamentous retraction, atrophy and dysplasia. The patient may have oedema and haematoma—usually, partial discontinuity of muscle fibres, bone remodelling, tedious calcification processes. − Risk factors: ergonomic, operational	Gradual Chronic inflammation Altered tissue Clearance needed Function change Pain therapy Functional rehabilitation

**Table 7:** Application of different types of classification to MSD recognised by ILO.

Musculoskeletal Disorders Name	Classification			
	ILO	ICD-10	MSD	
			Sudden	Gradual
Radial styloid tenosynovitis due to repetitive movements, intense efforts and extreme wrist postures	2.3.1	M65.4	2	3
Chronic tenosynovitis of the hand and wrist due to repetitive movements, intense efforts and extreme wrist postures	2.3.2	M65.8	-	3
Olecranon bursitis due to prolonged pressure in the elbow region	2.3.3	M70,2	2	-
Pre-patellar bursitis due to prolonged kneeling	2.3.4	M70.4	2	-
Repetitive and intense work epicondylitis	2.3.5	M77.1	2	3
Meniscus injuries after long periods of work in a kneeling or crouching position	2.3.6	M23.3	2	3
Carpal tunnel syndrome due to long periods of repetitive and intense work, work that involves vibration, extreme wrist postures or a combination of all three	2.3.7	G56.0	-	3

## Data Availability

Not applicable.
